# Visual Effect on Brain Connectome That Scales Feedforward and Feedback Processes of Aged Postural System During Unstable Stance

**DOI:** 10.3389/fnagi.2021.679412

**Published:** 2021-07-22

**Authors:** Yi-Ching Chen, Chien-Chun Huang, Chen-Guang Zhao, Ing-Shiou Hwang

**Affiliations:** ^1^Department of Physical Therapy, College of Medical Science and Technology, Chung Shan Medical University, Taichung, Taiwan; ^2^Physical Therapy Room, Chung Shan Medical University Hospital, Taichung, Taiwan; ^3^Department of Environmental and Occupational Health, National Cheng Kung University, Tainan, Taiwan; ^4^Department of Physical Therapy, College of Medicine, National Cheng Kung University, Tainan, Taiwan; ^5^Institute of Allied Health Sciences, College of Medicine, National Cheng Kung University, Tainan, Taiwan

**Keywords:** human aging, EEG, posture balance, graph analysis, visual input

## Abstract

Older adults with degenerative declines in sensory systems depend strongly on visual input for postural control. By connecting advanced neural imaging and a postural control model, this study investigated the visual effect on the brain functional network that regulates feedback and feedforward processes of the postural system in older adults under somatosensory perturbations. Thirty-six older adults conducted bilateral stance on a foam surface in the eyes-open (EO) and eyes-closed (EC) conditions while their center of pressure (COP) and scalp EEG were recorded. The stochastic COP trajectory was modeled with non-linear stabilogram diffusion analysis (SDA) to characterize shifts in postural control in a continuum of feedback and feedforward processes. The EEG network was analyzed with the phase-lag index (PLI) and minimum spanning tree (MST). The results indicated that visual input rebalanced feedforward and feedback processes for postural sway, resulting in a greater critical point of displacement (CD), short-term effective diffusion coefficients (D_s_) and short-term exponent (H_s_), but the smaller critical point of time (CT) and long-term exponent (H_l_) for the EC state. The EC network demonstrated stronger frontoparietal-occipital connectivity but weaker fronto-tempo-motor connectivity of the theta (4–7 Hz), alpha (8–12 Hz), and beta (13–35 Hz) bands than did the EO network. MST analysis revealed generally greater leaf fraction and maximal betweenness centrality (BC_max_) and kappa of the EC network, as compared with those of the EO network. In contrast, the EC network exhibited a smaller diameter and average eccentricity than those of the EO network. The modulation of long-term negative feedback gain of the aged postural system with visual occlusion was positively correlated with leaf fraction, BC_max_, and kappa, but negatively correlated with the diameter and average eccentricity for all EEG sub-bands. In conclusion, the aged brain functional network in older adults is tuned to visual information for modulating long-term negative feedback of the postural system under somatosensory perturbations.

## Introduction

During upright stance, sensory information from somatosensory, visual, and vestibular systems is integrated to regulate upright stance in a closed-loop fashion. Sensory degeneration has an interrelation effect on falling risk in older adults, who depend more strongly on visual input for postural control (Woollacott et al., 1986; Sundermier et al., 1996). Visual occlusion alters the internal reference of stance control, manifested as increases in irregularity and higher frequency components of the center of pressure (COP; Sabatini, 2000; King et al., 2020). By calculating the mean-squared COP displacements across various time intervals, stabilogram diffusion analysis (SDA) can yield additional insight into COP stochastic behaviors and underlying balance control mechanisms. The SDA plots of COP trajectories reveal two different postural behaviors between short- and long-time intervals. For the short-time intervals (typically less than 1s), the COP behaviors are persistent (positive correlation of COP data of the past and future), reflecting a predominant open-loop control on postural responses. In contrast, a closed-loop process prevails and results in anti-persistent postural responses with a negative correlation of COP data of the past and future. This stochastic model of SDA can quantify shifts in a continuum of the control regime ranging from feedforward (open-loop) to feedback (closed-loop) processes, in terms of changes in the transition between the short- and long-time intervals (or the critical point). The critical point specifies the time interval (critical time, CT) and sways amplitude (critical displacement, CD), beyond which postural sway behaviors are subject to the feedback process. With SDA, visual input is shown to modulate central feedforward [short-term diffusion coefficient (D_s_) /short-term scaling exponent (H_s_)] and reflex-lagged negative feedback mechanisms (long-term diffusion coefficient (D_l_)/long-term scaling exponent (H_l_); Collins and De Luca, 1993, 1995). Relative to the eyes-open (EO) state, the eyes-closed (EC) state enhances open-loop gain (D_s,_ H_s_) from the vestibular and proprioception afferents for posture control (Collins and De Luca, 1995; Doyle et al., 2008; Melzer et al., 2010). The EC state is also associated with a smaller D_l_ and H_l_ to regulate postural responses. With SDA, older adults (especially elderly fallers) were noted to favor a higher open-loop gain (D_s_) to regulate upright stance than did young adults (Amoud et al., 2007; Toosizadeh et al., 2015). In light of their greater critical point, older adults were less sensible to greater sway amplitude before the negative feedback mechanism was called into play for balance stabilization.

The power in localized EEG signals is tuned to the postural setting with and without access to vision. Eye closure during upright stance affects the brain states of all spectral distributions of EEG signals along the frontoparietal axis except the gamma band (>35 Hz; Thibault et al., 2014; Spironelli et al., 2016). Although no consensus has been reached on the specific frequencies subserving balance control, the potential linkage of the postural neural network to the visual subsystem has been a recent focus (Tewarie et al., 2014). During continuous balance, cortical theta activity in the fronto-central and centro-parietal regions increases with visually-induced postural threats (Mierau et al., 2017; Edwards et al., 2018), which is related to visual error detection and planning of corrective responses before falling consequences (Sipp et al., 2013). Concerning the visual information processing of the ventral and dorsal pathways (Mierau et al., 2017), alpha activity is most prominent in the occipital-parietal-temporal regions during stance with eyes closed. According to the gating function theory (Toscani et al., 2010), alpha activity is involved in the attentional process to suppress visual information transfer, and a transient “alpha drop” initiates thalamo-cortical information transfer for renewal of the balance state (Hülsdünker et al., 2016; Mierau et al., 2017). The EEG beta rhythm in the parietal and central cortical regions may contribute to the control of muscle synergy (Jacobs et al., 2015) and mediation of the information between the visual and sensorimotor systems (Peterson and Ferris, 2018; Solis-Escalante et al., 2019). Beta activity in the occipito-parietal areas is suppressed following visual perturbation onset, which is related to a decrease in motor inhibition to respond to sensorimotor conflicts (Peterson and Ferris, 2018; Malcolm et al., 2020). Although many previous studies have contrasted the regional cortical activities between the EO and EC states, little attention has been paid to the visual effect on the coordinated interplay of postural neural networks, especially in older adults who rely on visual input to prevent falls. More specifically, it still remains unclear how the brain mediates feedback and feedforward processes of the aged postural system with respect to visual inputs.

Visual occlusion on an unstable surface that can introduce conflicting sensory information is an effective intervention for geriatric balance rehabilitation. The aim of this study was to contrast bipedal stance on a foam surface in the EO and EC conditions in older adults, with a specific focus on the EEG–EEG functional connectivity of various sub-bands that underpin cortico-cortical interactions to regulate the feedback and feedforward processes of the postural system. To our knowledge, no previous studies have linked the vision-related shift in feedback–feedforward postural control to brain connectome contexts. This study hypothesized that visual input affects the relative significance of the feedforward and feedback processes to organize postural responses, as revealed by the degrees of neural integration and network configurations in a space. The present results may have applications in assessing degenerative balance dysfunction under sensory conflicts.

## Materials and Methods

### Subjects

For this study, 36 elderly participants over 60 years old (age: 66.1 ± 3.1, 15 males, 21 females) with regular exercise habits were recruited. They had normal or corrected-to-normal vision without known cognitive problems, history of falls, or diagnoses of neurological and musculoskeletal disorders requiring medication. This study was approved by an authorized institutional human research review board at the University Hospital (A-ER-107-099-T). All subjects signed consent forms prior to the experiment, in accordance with the Declaration of Helsinki.

### Experimental Procedures and Instrumentation

**Figure 1:**
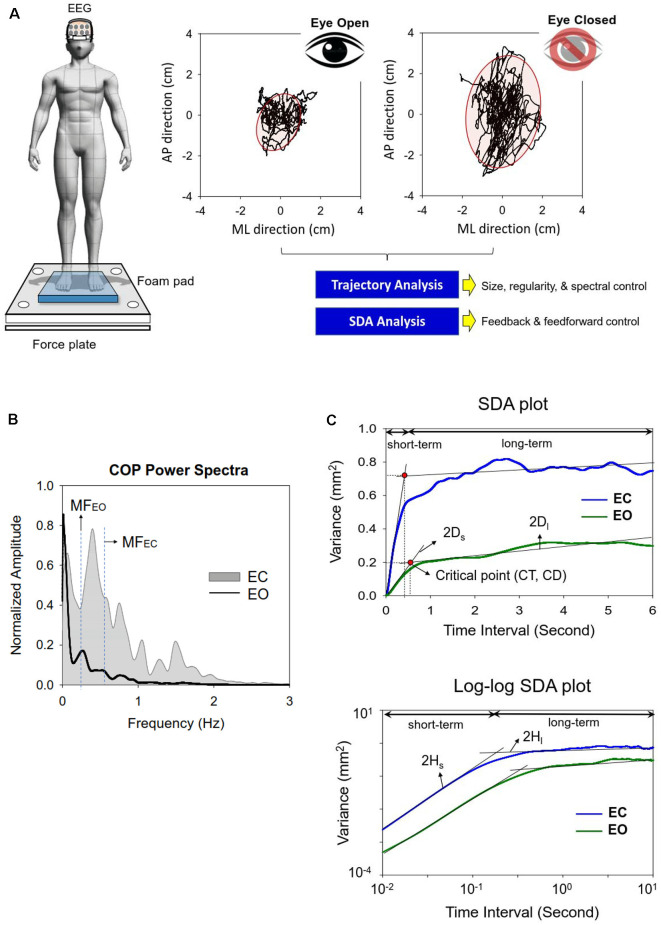
**(A)** Experimental setup for measurement of the dynamics of center of pressure (COP) and EEG signals in the eyes-open and eyes-closed conditions. **(B)** The contrasts the COP power spectra between the eyes-open (EO) and eyes-closed (EC) conditions from a typical subject. **(C)** Representative linear and log–log stabilogram diffusion (SDA) plots in the EO and EC conditions. Short-term and long-term regions fitted by straight black regression lines are dominated by the open-loop and closed-loop control strategies respectively. Stabilogram diffusion parameters (D_s_, D_l_, H_s_, H_l_) are determined by the slopes of lines fitted to short-term or long-term regions. A critical point is defined as the interaction point of the two regression lines in the short-term or long-term regions. CT, critical point of time; CD, critical point of displacement.

This study used a randomized, repeated measures design. On the day of the visit, the demographic data and health conditions of the participants were first gathered. Each participant completed an unstable postural task under the eyes-open (EO) and eyes-closed (EC) conditions. In the EO condition, the subjects needed to gaze at a fixed point on the wall and maintain for 60 s a steady bipedal stance with an inter-foot distance of one shoulder width on a foam surface (Airex Balance-pad, Switzerland; Figure 1A). Bilateral stance on the foam support with EO provided a stabilization effect on the altered proprioceptive and tactile information (Amoruso et al., 2011; Treger et al., 2020). In the EC condition, the participants were blindfolded with an opaque eye mask and maintained bilateral upright stance as in the EO condition. There were three trials of the two postural tasks with 3 min of rest between trials for each participant. The order of the EO and EC trials was randomized across subjects.

A 40-channel NuAmps amplifier (NeuroScan Inc., EI Paso, USA) with Ag-AgCl scalp electrodes was used to register fluctuations in scalp voltage during the postural tasks. Two electrodes were placed on the skin of the earlobes for offline re-referencing. After the scalp was rubbed with alcohol, conductive electrode gel was applied to the active electrode sites to improve electrode impedance. Scalp EEG signals were recorded from different cortical areas (Fp_1/2_, F_z_, F_3/4_, F_7/8_, FT_7/8_, FC_z_, FC_3/4_, C_z_, C_3/4_, CP_z_, CP_3/4_, P_z_, P_3/4_, T_3/4_, T_5/6_, TP_7/8_, O_z_, and O_1/2_), which were localized according to the international 10–20 system. For subtraction of eye movement and blink artifacts, horizontal electrooculography (EOG) data were collected with two electrodes placed at the outer canthus of the left and right eyes. For off-line vertical EOG assessment, two electrodes were placed infra- and supra-orbitally at the right eye, respectively. The impedances of all the electrodes were below 5 kΩ and were referenced to linked mastoids of both sides. The EEG data were recorded with a band-pass filter set at 0.1–100 Hz and a sampling rate of 1 kHz. Synchronized with scalp EEG signals, a force plate (Kistler Type 9260A, Switzerland) was used to record the trajectory of postural sway during bilateral stance in the EO and EC conditions. The force plate data were then conditioned with an amplifier (DAQ for BioWare Type 5695B, Switzerland) and sampled at a rate of 1 kHz using BioWare^®^ software (Type 2812A, Switzerland).

### Data Analysis

#### Traditional Analysis of Center of Pressure

A set of traditional COP metrics were analyzed, including: (1) 95% confidence ellipse sway area (CEA—unit in cm^2^), (2) mean frequency (MF), and (3) sample entropy (SampEn). For data stability, the COP data were analyzed from the 3rd second to the 58th second after low-pass filtering (cut-off frequency: 6 Hz). Mean frequency (MF) was estimated from the power spectra of detrended COP time-series, or the distance of each COP data point from the sway center, followed by removal of a linear trend. Power spectra were estimated using a fast Fourier transform and the Welch method (Hanning window, window length: 15 s, overlapping time segment: 25% × window length, spectral resolution: 0.02 Hz; Figure 1B). The COP data were down-sampled to 100 Hz for sample entropy (SampEn) calculation, as in previous studies (Lubetzky et al., 2018). The mathematical formula of sample entropy was $SampEn(m,r,N)=-\log\left(\sum_{i=1}^{N-m}A_{i}/\sum_{i=1}^{N-m}B_{i}\right),$ where *r* = 20% of the standard deviation of the data, *m* is the length of the template (*m* = 2), and *N* is the number of data points in the time series. *A*_i_ is the number of matches of the *i*th template of length *m* + 1 data points, and *B*_i_ is the number of matches of the *i*th template of length *m* data points. Postural sway regularity reflects attentional investment in postural control, and an increase in the regularity (or smaller SampEn) of sway response correlates to more attentional investment to postural control (Roerdink et al., 2011).

#### Stabilogram Diffusion Analysis of Center of Pressure

SDA was used to characterize the shift in feedback and feedforward controls of the COP (Collins and De Luca, 1993, 1995), which is not possible with the use of traditional metrics. SDA is a probabilistic tool that describes the power-law relationship between the mean-squared value (or variance) of detrended COP time-series (<*dCOP*^2>^) and the time interval (*dt*) in which these values occur. SDA was calculated with the following equation: $\langle dCOP^{2}\rangle =\langle {\left[x(t+dt)-x(t)\right]}^{2}\rangle ,$ where <•> indicates the mean of the detrended COP time-series. The computation of *dCOP*^2^ was repeated with increasing *dt* values ranging from 0 to 6 s. The diffusion plot (linear–linear plots or log–log plots) was the mean square of the detrended time-series of the COP data <*dCOP*^2^ against the time intervals *dt* (Figure 1C). The critical point of time (CT) was the intersection of the two regression lines of the linear–linear diffusion plot, and variations in the critical point of displacement (CD) reflected a paradigm shift in the amount of COP sway (Collins and De Luca, 1993, 1995; Figure 1C). The critical point estimates the transition from predominantly open-loop to predominantly closed-loop control. A greater CD indicates that larger postural sway takes place before closed-loop control begins to predominate postural sway behaviors. A greater CT reflects a longer lag time for the posture system to engage in closed-loop control mechanism. In the linear–linear diffusion plot, the regression slopes (D_s_ and D_l_) of the short-term and long-term regions were two effective diffusion coefficients, which parameterized the control of the force stochastic activities in those regions, respectively. The short-term and long-term scaling exponents (H_s_ and H_l_) were linear fits of the log–log plot of the SDA. A scaling exponent greater than 0.5 indicates that the system is governed by the open-loop process and that the data series of the past and future are positively correlated with the persistence property (Collins and De Luca, 1993, 1995). A greater H_s_ or D_s_ represented a larger open-loop gain for postural regulation. Conversely, a scaling exponent smaller than 0.5 indicates that the data series of the past and future are negatively correlated with the anti-persistence property, as regulated by the closed-loop process. A smaller H_l_ or D_l_ represented a larger closed-loop gain for postural regulation. The COP variables were analyzed in MATLAB R2019a software (Mathworks, USA).

#### EEG Inter-regional Connectivity and Minimum Spanning Tree Analysis

All the EEG data were first filtered between 1 and 60 Hz using a zero-phase finite impulse response (FIR) filter (60 dB/octave) to remove the DC shift. The blinks were detected by creating a bipolar vertical EOG channel by subtracting activity in the infraorbitally-placed electrode from that in the superorbitally-placed electrode. Correction of ocular artifacts was performed with the NeuroScan 4.3 software program (NeuroScan Inc., EI Paso, TX, USA), based on regression analysis (Semlitsch et al., 1986). In line with the COP data, the EEG signals of the first and last 2 s were not analyzed. The EEG data of part of the run were segmented in 2 s epochs. To further confirm valid artifact-free EEG epochs, all epochs surviving automated artifact rejection were visually inspected for undetected artifacts by the researchers. The phase-lag index (PLI) and variables of minimum spanning trees (MSTs) were used to analyze artifact-free epochs to sub-bands of the EEG data of the delta (1–3 Hz), theta (4–7 Hz), alpha (8–12 Hz), and beta (13–35 Hz) bands. Functional connectivity between the EEG time-series of all 30 electrode pairs was calculated with the phase-lag index (PLI), which is insensitive to volume conduction (Stam et al., 2007; Tewarie et al., 2014). Computation of the PLI across all pairs of sub-band EEG data of the channels produced a square 30 × 30 PLI adjacent matrix (such as the alpha adjacent matrix in the 8–12 Hz band). The PLI indexes the distribution asymmetry of phase differences in the instantaneous phases of two time series, derived from the Hilbert transformation. If φ(*t*) is the phase difference, the PLI is defined thus: PLI=|E{sgn(Δφ(t))}|, where *sgn* is a function that extracts the sign of a real number. PLI measure has the advantage of minimizing bias from common sources (such as volume conduction) for EEG and MEG measures (Stam et al., 2007). The network topology of the PLI functional connectivity matrix was characterized by MSTs (Stam et al., 2014). To highlight the core properties of the functional network, an MST simply includes the strongest connections of all nodes of the functional network without inter-connection loops (Stam et al., 2014; Chen et al., 2021). MST can effectively underscore the core properties of the information flow within the EEG connectome by including the high-probability connections of all the shortest paths without loops in the network (Stam et al., 2014; van Diessen et al., 2015). MST is a cost-effective method with test–retest reliability for featuring major traffic in a weighted network, with statistical rigor for unequal sizes of network nodes in different experimental conditions (Mandke et al., 2018; van Dellen et al., 2018). MST variables are sensitive to small network differences (van Diessen et al., 2015) without the arbitrary selection of connectivity thresholds used in the traditional graph-based analysis (Tewarie et al., 2014; Varghese et al., 2019).

**Figure 2:**
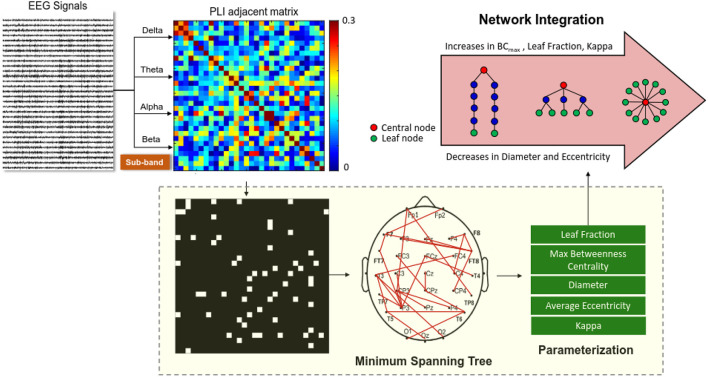
Schematic representation of network integration using variables of EEG-based minimum spanning trees (MSTs). A functional connectivity matrix of the preprocessed sub-band EEG (delta, theta, alpha, or beta rhythms) was constructed using a phase-lag index. MST is a binary backbone graph (edge weights 0 or 1) that includes the strongest connections of the functional connectivity matrix without loops. Five important variables (leaf fraction, maximal betweenness centrality (BC_max_), diameter, average eccentricity, and kappa) were selected from the MSTs to index brain network properties.

Figure 2 summarizes a calculation pipeline of an MST from artifact-free EEG epochs. Five key graph measures of the MST (diameter, leaf fraction, average eccentricity, maximal betweenness centrality (BC_max_), and kappa) were used to describe the level of network integration. The definitions of these measures are provided in Table 1. These MST variables, estimated from sub-band EEG signals of three experimental trials, were averaged for each subject. The PLI functional connectivity was calculated with the HERMES function in Matlab (Niso et al., 2013). Parameterization of the MST and network properties was accomplished with functions of the Brain Connectivity Toolbox (Rubinov and Sporns, 2010).

**Table 1:** Definitions of five minimal spanning tree (MST) variables for EEG brain topology.

MST Measures	Definition	Correlation
Leaf Fraction	The ratio of the number of nodes that have only one edge.	Positive correlation to network integration.
Diameter	The largest distance between any two nodes normalized for the total number of connections.	Negative correlation to network integration.
Average Eccentricity	The shortest-path distances between node I and any other tree node, average eccentricity being the mean value of all nodes.	Negative correlation to network integration.
BC_max_	The highest value of betweenness centrality in the network, betweenness centrality being a measure of centrality in a graph based on shortest paths.	Positive correlation to network integration.
Kappa	Measure of the broadness of the degree distribution.	Positive correlation to network integration.

### Statistical Analysis

The connectivity strength within the PLI adjacent matrix in the EO and EC conditions was contrasted with paired *t*-tests. A set of supra-threshold connections (|*t*_35_| > 3.340, *p* < 0.001) was extracted to highlight the differences in topological distributions between the EO and EC conditions. Multi-variate Hotelling’s T-squared statistics were used to examine the visual effect (EO vs. EC) on the COP trajectory characteristics (CEA, MF, and SampEn), SDA variables (CT, CD, D_s_, D_l_, H_s_, and H_l_), and MST variables (leaf fraction, diameter, average eccentricity, BC_max_, and kappa) of different sub-bands. The *post hoc* test was the Bonferroni test. The significances of the correlations between the SDA and MST variables in the EO and EC conditions were examined with Pearson’s correlation. Of particular interest is that the relationship between brain network reorganization and shift in postural control between the feedforward and feedback processes was examined with Pearson’s correlation between normalized differences (ND) in the SDA and MST variables in the EO and EC conditions (ND = (EC − EO)/EO). Data are presented as group means ± standard deviation. All statistical analyses were performed in IBM SPSS Statistics (v19). The level of significance was 0.05.

## Results

### Center of Pressure and SDA

Table 2 shows the results of Hotelling’s T-squared statistics to contrast the COP characteristics between the EO and EC conditions using traditional analysis. The COP trajectory characteristics differed between the two visual conditions (Wilks’ *Λ* = 0.181, *p* < 0.001, $\eta_{p}^{2}$ = 0.819). EC resulted in greater CEA, MF, and SampEn than did EO (*p* < 0.001). Table 3 summarizes the results of Hotelling’s T-squared statistics to contrast the SDA variables between the EO and EC conditions. The SDA variables were significantly different between the EO and EC conditions (Wilks’ *Λ* = 0.106, *p* < 0.001, $\eta_{p}^{2}$ = 0.894). *Post hoc* analysis revealed that CT and H_l_ were smaller in the EC condition than in the EO condition (*p* < 0.001). CD and D_s_, and H_s_ were greater in the EC condition than in the EO condition (*p* < 0.001).

**Table 2:** The contrast population means of center-of-pressure (COP) variables between the eyes-open (EO) and eyes-closed (EC) conditions.

COP Trajectory	EO	EC	Hotelling’s Statistics	*Pos hoc* test
**CEA** (cm^2^)	3.670 ± 2.180	9.343 ± 5.779	Wilks’ *Λ* = 0.181, *p*< 0.001	*t*_35_ = 8.641, *p*< 0.001, $\eta_{p}^{2}$ = 0.681
**MF** (Hz)	0.454 ± 0.104	0.670 ± 0.143	$\eta_{p}^{2}$ = 0.819	*t*_35_ = 7.830, *p*< 0.001, $\eta_{p}^{2}$ = 0.637
**SampEn**	0.139 ± 0.031	0.184 ± 0.045		*t*_35_ = 8.209, *p*< 0.001, $\eta_{p}^{2}$ = 0.658

*CEA: 95% confidence ellipse sway area; MF, mean frequency; SampEn, sample entropy*.

**Table 3:** The contrast population means of variables of stabilogram diffusion analysis (SDA) between the eyes-open (EO) and eyes-closed (EC) conditions.

SDA Analysis	EO	EC	Hotelling’s Statistics	*Pos hoc* test
**CT** (s)	0.736 ± 0.184	0.562 ± 0.090		*t*_35_ = 5.980, *p*< 0.001, $\eta_{p}^{2}$ = 0.505
**CD** (mm^2^)	0.252 ± 0.125	0.860 ± 0.405		*t*_35_ = −11.502, *p*< 0.001, $\eta_{p}^{2}$ = 0.791
**D_s_** (mm^2^/s)	0.183 ± 0.086	0.789 ± 0.328	Wilks’ *Λ* = 0.106, *p*< 0.001	*t*_35_ = −12.947, *p*< 0.001, $\eta_{p}^{2}$ = 0.827
**D_l_** (mm^2^/s)	0.005 ± 0.004	0.006 ± 0.006	$\eta_{p}^{2}$ = 0.894	*t*_35_ = −1.171, *p* = 0.249, $\eta_{p}^{2}$ = 0.038
**H_s_** (mm^2^/s)	0.853 ± 0.034	0.893 ± 0.024		*t*_35_ = −7.694, *p*< 0.001, $\eta_{p}^{2}$ = 0.628
**H_l_** (mm^2^/s)	0.071 ± 0.036	0.023 ± 0.029		*t*_35_ = 4.999, *p*< 0.001, $\eta_{p}^{2}$ = 0.417

*CT, critical point of time; CD, critical point of displacement; D_s_, short-term effective diffusion coefficients; D_l_, long-term effective diffusion coefficients; H_s_, short-term scaling exponent; H_l_, long-term scaling exponent*.

### EEG Functional Connectivity and MST

**Figure 3:**
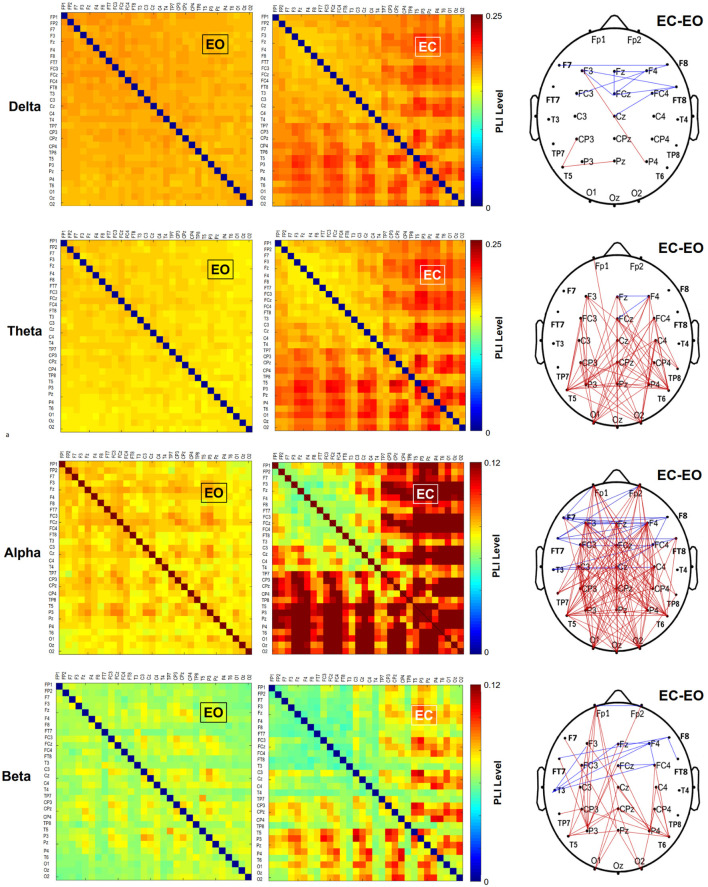
The pooled adjacent matrix of the phase-lag index (PLI) of artifact-free EEG epochs for bilateral stance in the EO and EC conditions in different sub-bands. Paired *t*-tests were independently performed in a PLI matrix (30 × 30) to examine the differences in EEG connectivity of each sub-band between the EO and EC conditions. Only those t-statistics larger or smaller than an uncorrected threshold (|*t*_35_| > 3.340, *p* < 0.001) are considered as supra-threshold connections. Contrasting wiring diagrams on the scalp (the right plots) show the topological distributions of the sub-band suprathreshold connectivity that differ with and without visual input (EO vs. EC). The visual effects are band dependent, resulting in selective enhancement and depression of EEG connectivity (Red line: EC supra-threshold connectivity > EO supra-threshold connectivity, *p* < 0.001; blue line: EO supra-threshold connectivity > EC supra-threshold connectivity, *p* < 0.001).

The functional connectome at the four sub-bands for bilateral stance with EO and EC was characterized with a PLI adjacent matrix (Figure 3, left and middle plots). The visual effect on inter-regional connectivity was highlighted with the scalp topology of the supra-threshold connectivity between the EO and EC conditions (|*t*_35_| > 3.340, *p* < 0.001). The difference in inter-regional connectivity due to the visual effect was most evident in the alpha band. Compared to the EO condition, the EC condition generally exhibited weaker functional connectivity in the fronto-tempo-motor network of the bilateral hemispheres and stronger frontoparietal-occipital connectivity, especially in the theta, alpha, and beta bands. Tables s 4A–D summarize the results of Hotelling’s T-squared statistics to compare the sub-band MST variables between the EO and EC conditions. The MST variables varied with the EO and EC conditions for all sub-band connectivity (Delta: Wilks’ *Λ* = 0.703, *p* = 0.043, $\eta_{p}^{2}$ = 0.297; Theta: Wilks’ *Λ* = 0.536, *p* = 0.001, $\eta_{p}^{2}$ = 0.464; Alpha: Wilks’ *Λ* = 0.440, *p* < 0.001, $\eta_{p}^{2}$ = 0.560; Beta: Wilks’ *Λ* = 0.566, *p* = 0.002, $\eta_{p}^{2}$ = 0.434). Leaf fraction, BC_max_, and kappa tended to be greater in the EC condition than in the EO condition across different sub-bands (*p* < 0.001 to *p* < 0.017), except for kappa in the delta band (*p* = 0.058). In contrast, the diameter and average eccentricity were smaller in the EC condition than in the EO condition across the different sub-bands (*p* ≤ 0.015).

**Table 4:** The contrast population means of network integration graph measures between the eyes-open (EO) and eyes-closed (EC) conditions.

(A)
Delta (1–3 Hz)	EO	EC	Hotelling’s Statistics	*Pos hoc* test
**Leaf Fraction**	0.544 ± 0.021	0.564 ± 0.047		*t*_35_ = 2.574, *p* = 0.014, $\eta_{p}^{2}$ = 0.159
**Diameter**	0.344 ± 0.125	0.331 ± 0.028		*t*_35_ = −2.596, *p* = 0.014, $\eta_{p}^{2}$ = 0.161
**Ave. Ecc**	0.271 ± 0.011	0.262 ± 0.021	Wilks’ *Λ* = 0.703, *p* = 0.043	*t*_35_ = −2.551, *p* = 0.015, $\eta_{p}^{2}$ = 0.157
**BC_max_**	0.717 ± 0.011	0.729 ± 0.032	$\eta_{p}^{2}$ = 0.297	*t*_35_ = 2.234, *p* = 0.026, $\eta_{p}^{2}$ = 0.134
**Kappa**	3.128 ± 0.151	3.376 ± 0.747		*t*_35_ = 1.962, *p* = 0.058, $\eta_{p}^{2}$ = 0.099
(B)
Theta (4–7 Hz)	**EO**	**EC**	Hotelling’s Statistics	*Pos hoc* test
**Leaf Fraction**	0.545 ± 0.020	0.571 ± 0.048		*t*_35_ = 3.549, *p* = 0.001, $\eta_{p}^{2}$ = 0.265
**Diameter**	0.343 ± 0.014	0.327 ± 0.027		*t*_35_ = −3.462, *p* = 0.001, $\eta_{p}^{2}$ = 0.255
**Ave. Ecc**	0.270 ± 0.010	0.258 ± 0.021	Wilks’ *Λ* = 0.536, *p* = 0.001	*t*_35_ = −3.523, *p* = 0.001, $\eta_{p}^{2}$ = 0.262
**BC_max_**	0.719 ± 0.012	0.734 ± 0.035	$\eta_{p}^{2}$ = 0.464	*t*_35_ = 2.575, *p* = 0.014, $\eta_{p}^{2}$ = 0.159
**Kappa**	3.133 ± 0.150	3.467 ± 0.809		*t*_35_ = 2.511, *p* = 0.017, $\eta_{p}^{2}$ = 0.153
(C)
Alpha (8–12 Hz)	**EO**	**EC**	Hotelling’s Statistics	*Pos hoc* test
**Leaf Fraction**	0.545 ± 0.022	0.587 ± 0.050		*t*_35_ = 5.471, *p*< 0.001, $\eta_{p}^{2}$ = 0.456
**Diameter**	0.341 ± 0.014	0.315 ± 0.028		*t*_35_ = −5.694, *p*< 0.001, $\eta_{p}^{2}$ = 0.481
**Ave. Ecc**	0.268 ± 0.011	0.249 ± 0.021	Wilks’ *Λ* = 0.440, *p*< 0.001	*t*_35_ = −5.760, *p*< 0.001, $\eta_{p}^{2}$ = 0.487
**BC_max_**	0.717 ± 0.013	0.744 ± 0.034	$\eta_{p}^{2}$ = 0.560	*t*_35_ = 4.665, *p*< 0.001, $\eta_{p}^{2}$ = 0.383
**Kappa**	3.121 ± 0.177	3.638 ± 0.815		*t*_35_ = 3.994, *p*< 0.001, $\eta_{p}^{2}$ = 0.308
(D)
Beta (13–35 Hz)	**EO**	**EC**	Hotelling’s Statistics	*Pos hoc* test
**Leaf Fraction**	0.538 ± 0.025	0.564 ± 0.038		*t*_35_ = 3.861, *p*< 0.001, $\eta_{p}^{2}$ = 0.299
**Diameter**	0.347 ± 0.016	0.331 ± 0.023		*t*_35_ = −4.159, *p*< 0.001, $\eta_{p}^{2}$ = 0.331
**Ave. Ecc**	0.273 ± 0.012	0.260 ± 0.017	Wilks’ *Λ* = 0.566, *p* = 0.002	*t*_35_ = −4.189, *p*< 0.001, $\eta_{p}^{2}$ = 0.334
**BC_max_**	0.714 ± 0.013	0.727 ± 0.024	$\eta_{p}^{2}$ = 0.434	*t*_35_ = 3.482, *p* = 0.001, $\eta_{p}^{2}$ = 0.257
**Kappa**	3.088 ± 0.187	3.333 ± 0.512		*t*_35_ = 2.831, *p* = 0.008, $\eta_{p}^{2}$ = 0.186

*Ave. Ecc: average eccentricity; BC_max_: maximal betweenness centrality*.

### Correlations Between SDA and MST

**Figure 4:**
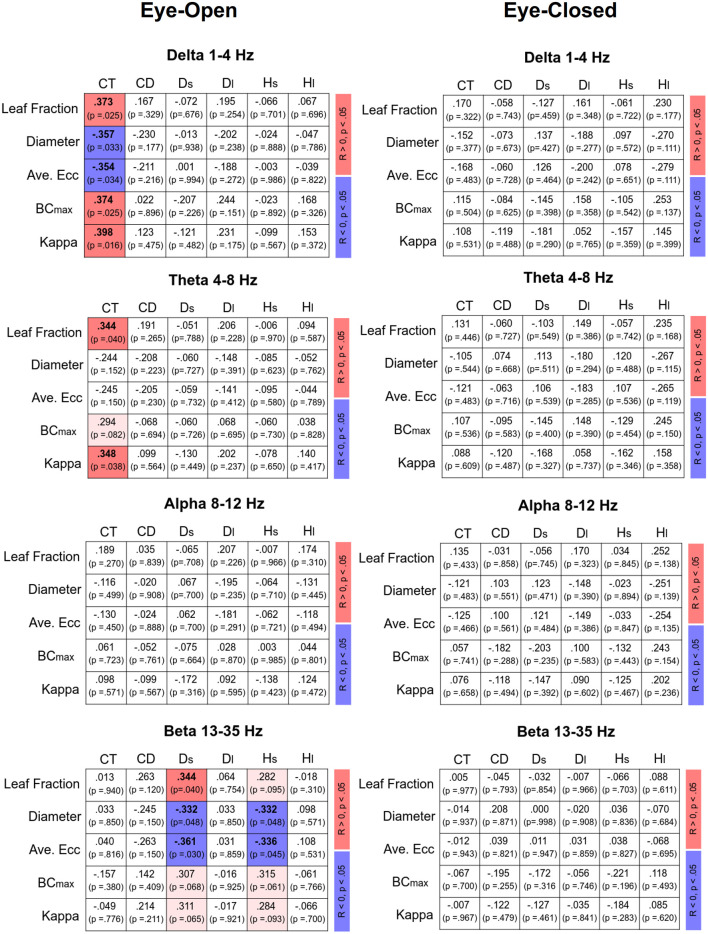
Correlation matrix of sub-band MST and SDA variables and corresponding significance levels for the eyes-open (EO) and eyes-closed (EC) conditions. Red and blue shaded areas represent significant positive and negative correlations (*p* < 0.05) of the sub-band MST and SDA variables, respectively. Light shaded areas represent marginally significant correlation of the sub-band MST and SDA variables (*p* < 0.10).

**Figure 5:**
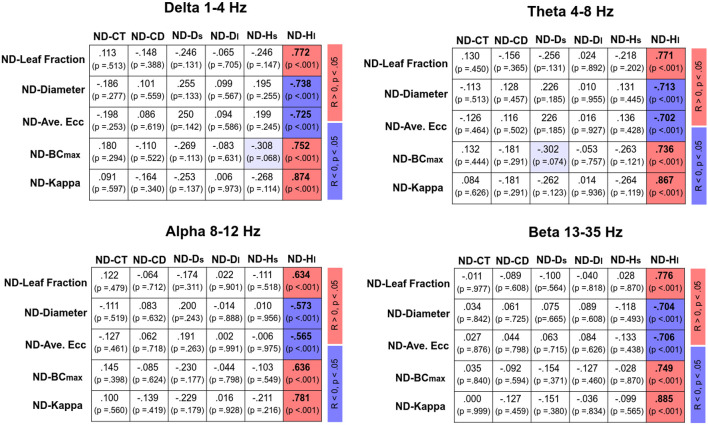
Correlation matrix of normalized difference in sub-band MST and normalized difference in SDA variables [normalized difference (ND): (EO − EC)/EC]. Red and blue shaded areas represent significant positive and negative correlations (*p* < 0.05) of the normalized difference in sub-band MST and normalized difference in SDA variables, respectively. Light shaded areas represent marginally significant correlation of the sub-band MST and SDA variables (*p* < 0.10).

Figure 4 displays Pearson’s correlations of SDA and sub-band MST variables in the EO and EC conditions, respectively. In the EO condition, CT was positively correlated with the leaf fraction, BC_max_, and kappa of the delta band (*p* < 0.05). In addition, CT was positively correlated with the leaf fraction and kappa of the theta band (*p* < 0.05). However, CT was negatively correlated with the diameter and average eccentricity of the delta band (*p* < 0.05). D_s_ was positively correlated with the leaf fraction of the beta band, while D_s_ and H_s_ were negatively correlated with the diameter and average eccentricity of the beta band (*p* < 0.05). In contrast, SDA and sub-band MST variables were not significantly correlated in the EC condition (*p* > 0.05). Figure 5 summarizes the Pearson’s correlations of normalized differences in the SDA (ND-SDA) and sub-band MST (ND-MST) variables between the EO and EC conditions. Significant correlations were consistently found between ND-H_l_ and normalized differences in all sub-band MST measures (*p* < 0.001). ND-H_l_ was positively correlated with ND-leaf fraction, ND-BC_max_, and ND-Kappa (*p* < 0.001), but negatively correlated with ND-diameter, and ND-average eccentricity (*p* < 0.001). This fact suggested that the visual effect on the feedback process was dependent on variation in the cortical network integration.

## Discussion

### Variations in Feedback–Feedforward Control of Postural Sway Due to Visual Input

In light of the CEA and mean frequency of the COP trajectory (Table 2), it appears that eye closure reduces the available sensory information for maintaining balance, resulting in greater postural sway and stance uncertainty with increasing correction attempts during the foam stance. In addition, EC added to the complexity of COP trajectory with higher SampEn than EO, in accordance with previous observations in the literature (Borg and Laxåback, 2010; da Costa Barbosa and Vieira, 2017). In contrast to young adults, who did not exhibit complexity increase in COP trajectory with visual occlusion, this observation would suggest that the elderly favor proprioceptive over visual information for autonomous postural control in the EC state (da Costa Barbosa and Vieira, 2017). In fact, eye closure increases the use of alternative sensory modalities for balance control and thus could alter open- and closed-loop postural control behaviors. Our visual effect on the SDA results of older adults standing on a foam surface was consistent with previous studies, which showed a shift in the control scheme of the feedforward and feedback processes in healthy young adults (Collins and De Luca, 1995) and in patients with vertigo (Wuehr et al., 2013) on a firm surface. As compared with the EO condition, the shorter critical point of time (CT) of the EC condition indicated an early transition from open-loop control to closed-loop control due to the inability of the pre-programmed feedforward process to regulate an unexpected postural disturbance. Known as control sensitivity loss, the greater critical point of displacement revealed that the feedback control was called into play to stabilize the posture response only when postural fluctuations were larger. In addition, the EC state led to a smaller long-term scaling exponent (H_l_) than that of EO, suggesting that the COP data in the long-term region became more negatively correlated for the EC condition (Collins and De Luca, 1995). Hence, negative feedback gain of the CNS was exaggerated for postural control following visual occlusion. Theoretically, the feedback control with time delays and exaggerated loop gain could significantly undermine the stability of the postural system, which may explain age-related decline in postural control, especially for elderly fallers (Melzer et al., 2010) and patients with neurological disorders (Wuehr et al., 2013; Toosizadeh et al., 2015; Treger et al., 2020).

### Visual Impact on EEG Sub-band Postural Network

Previous studies reported significant differences in the states of cortical activation for the unavailability of visual input, including increases in theta (4–7 Hz), alpha (8–12 Hz), and beta (13–35 Hz) rhythms to reconcile increasing sensory conflicts with EC (Varghese et al., 2015, 2019). In the EC situation, immediate theta enhancements in the anterior cingulate and anterior parietal cortex (Sipp et al., 2013; Ozdemir et al., 2018) were hypothesized to be linked with the initiation of appropriate postural responses and the detection of postural threats (Slobounov et al., 2009). The widespread alpha rhythm during upright stance with EC is referred to as inhibitory control of information processing from task-irrelevant areas, according to the “gating function theory.” When visual input is available, alpha oscillations desynchronize, which makes allowances for intra- and extrapersonal space information with postural memories from the visual system (Del Percio et al., 2007). A power decrease in the beta band throughout the gait cycle was mostly reported in walking balance control (Yokoyama et al., 2021). The prevailing explanation for the decrease in the beta rhythm is the detection of a change in the status quo and termination of the current motor action (Peterson and Ferris, 2018). During standing, the visual optic flow produces more pronounced beta desynchronization, corresponding to preparation for and execution of postural adjustments against stance destabilization (Malcolm et al., 2018, 2020).

The analysis of the sub-band inter-regional connectivity provided additional insight into unstable foam stance with EC. In comparison with EO, EC increased the long–range connectivity between the frontoparietal-occipital areas in the theta, alpha, and beta bands (Figure 3). This stronger long–range connectivity may reflect the enhancement of frontal attempts to detect postural errors from peripheral sensory channels to regain balance (theta rhythm) in consequence to idle processing of the dorsal visual streams (Peterson and Ferris, 2019), which convey information about the spatial orientation from the occipital area (alpha rhythm). Consistent with this interpretation is that beta synchronization of the long–range connectivity with EC reflects a decrease in intentional postural adjustments with ample sensory feedback, despite the increasing postural sway. In contrast, connectivity within the fronto-central network for all sub-bands waned with vision occlusion. During the unstable foam stance, the EC-related desynchronization could have been involved with releases of the frontal eye field (FEF) to control ocular movement and the fronto-insular–temporal network, which receives body-related visual input from extrastriate visual areas for planning the postural configuration (Amoruso et al., 2011; Zimmermann et al., 2018). Interestingly, the distinct inter-regional modulation for the unstable foam stance with EC was largely consistent with the affordance competition hypothesis for visually-guided movement, which dichotomizes brain networks into processes of action specification (the visual dorsal stream) and action selection (the frontal/prefrontal and basal ganglia loops; Cisek, 2007; Cisek and Kalaska, 2010). Consistent with this hypothesis, during the foam surface stance with EC, the dorsal stream that transforms visual information into representations of intended actions was relatively idle. During visual occlusion, EC-related decreases in connectivity within the fronto-central network reflected less sensory information pertinent to action selection to be processed by the frontal/prefrontal areas. However, it is hard to link the topological variations in sub-band supra-connectivity to the visual effect on postural control in the context of feedback and feedforward processes.

### Functional Linkage Between EEG MST and Feedback–Feedforward Control

The use of MST analysis to parameterize the core information flow within the EEG connectome is helpful to link with postural control in a continuum of feedback and feedforward processes. In the EO condition, the critical point of time (CT) was related to MST variables in the delta and theta bands, whereas the short-term diffusion coefficient (D_s_) and short-term scaling exponents (H_s_) were significantly related to MST variables in the beta band (Figure 4; left). Given the consistent correlations between SDA modeling and EEG MST networks, this led to a novel finding of a “cortical balance network” engaged in shifting between feedforward and feedback processes with specific brain oscillations. When visual information was available during the foam stance, CT increased (in favor of the feedforward process for postural control for more time intervals) in proportion to the higher degree of network integration (greater leaf fraction, BC_max_, and kappa; smaller diameter and average eccentricity) in the delta and/or theta bands. Compared to healthy counterparts, fall-prone patients with a shorter CT tend to have some limitations in short-term, open-loop control, which may lead to maladaptive responses to postural destabilization (Wuehr et al., 2013; Toosizadeh et al., 2015). Fall-prone older adults are therefore thought to have impaired network integration in the delta and/or theta sub-bands. Functionally, cortical representations of the delta band are thought to be linked with planning and executing coordinated multi-joint movements, especially for kinematics in the lower limbs during posture and locomotion (Ozdemir et al., 2018). The frontal theta rhythm is related to postural error detection from the anterior cingulate cortex and sensorimotor areas to initiate proper postural responses to cope with postural instability (Sipp et al., 2013; Hülsdünker et al., 2015; Mierau et al., 2017). On the other hand, the short-term diffusion coefficient (D_s_) and scaling exponent (H_s_) were correlated with the MST beta variables (Figure 4). On account of the negative correlations, decreases in diameter and average eccentricity were associated with increases in H_s_ and D_s_, which are ordinary SDA observations for fall-prone patients (Wuehr et al., 2013; Toosizadeh et al., 2015; Treger et al., 2020). Hence, increasing the integration demands with a smaller diameter and average eccentricity in the beta MST network indicates a persistent status quo for stance control, which is unfavorable for standing on an unstable foam surface. Older adults with greater beta suppression in the midfrontal and parietal regions showed less postural sway during tandem stance with optic flow manipulations (Malcolm et al., 2020). In practice, beta suppression could be a cortical marker of a flexible postural strategy tuned to environmental changes.

Despite the higher level of network integration of all the EEG sub-bands in the EO condition (Tables s 4A–D), none of the MST variables were significantly correlated with SDA variables (Figure 4, right). The insignificance indicates that the enhanced network integration was not responsible for modulation of the feedforward–feedback control during the unstable upright stance with EC. To maintain an upright stance, information gathered from all sensory channels is finally sent to the cortex for perception and processing (Takakusaki, 2017). When the elderly stand on a foam surface with EC, sensory conflicts are accentuated without the moderating effect of visual input to reconcile cortical sensory conflict (Anacker and Di Fabio, 1992; Buatois et al., 2007). It is, therefore, speculated that the network integration of different EEG bands might be relevant to solving the emerging problem of sensory integration caused by vestibular and proprioceptive conflicts. Under the EC condition, active maintenance of postural balance with feedforward and feedback processes could mainly be achieved by subcortical structures, such as the cerebellum, basal ganglia, and brainstem regions (Zenzeri et al., 2014; Takakusaki, 2017). Closely cooperating with the basal ganglia, the cerebellum, receiving an efference copy of a central motor command, and proprioceptive and vestibular afferents, is a center for predicting sensory events and associated posture commands with novel sensory consequences (Horak and Diener, 1994). The anticipatory postural adjustments regulate postural command *via* the cortico-reticular, vestibulospinal and reticulospinal tracts (Takakusaki, 2017), which explains the insignificant correlation between a shift in the postural control feedforward process with EC and the cortical network integration in the elderly.

### Visual Effect on Correlation of Postural Neural Network and Feedback–Feedforward Control

In terms of MST variables, the changes in the network integration of all EEG sub-bands nicely predicted the visual effect on the long-term scaling exponent (H_l_) from EO to EC (Figure 5). When visual information was occluded, a higher degree of network integration was associated with an occlusion-related decrease in H_l_. The reorganization of the cortical network due to eye closure was to tune the anti-persistent behavior of the postural sway *via* negative closed-loop mechanisms of the postural system. This fact lends neurophysiological support to a hypothetical model proposed by Collins and De Luca (1995), which posits that visual inputs modulate the operational characteristics of the closed-loop postural control from the proprioceptive and/or vestibular afferents (11). On account of the highly significant correlation between normalized changes in network integration and the long-term scaling exponent, visual input has a potent impact on the postural feedback systems of older adults, a finding which is compatible with empirical observations that older adults rely preferentially on the vision for posture control (Thibault et al., 2014; Spironelli et al., 2016) and that accidental falls occur in this population when vision and proprioception are simultaneously challenged (Lord and Menz, 2000; Anson et al., 2019).

## Conclusion

The present study was the first to attempt to connect the visual effect on the feedforward–feedback processes of posture control to cortical networks in older adults during an unstable stance. As compared to the EC state, the EO state exhibits stronger fronto-central connectivity and weaker frontoparietal-occipital connectivity in the theta, alpha, and beta bands. In the EO state, the critical time point for shifting to feedforward and feedback processes in an aged postural system is dependent largely on the network integration of delta and theta oscillations. A greater critical time point is associated with greater leaf fraction and kappa in the delta and theta bands, in addition to maximal betweenness centrality in the delta band. In contrast, a greater critical time point is associated with the smaller diameter and average eccentricity in the delta band. The short-term feedforward gain of the aged postural system is negatively correlated with the diameter and average eccentricity of the beta network. In the EC condition, integration of the cortical network was independent of the regulation of the feedforward and feedback processes of the postural system. From EO to EC, changes in the integration of the postural neural network of all sub-bands well predict gain modulation of the negative feedback of older adults while they stand on an unstable surface. Visual occlusion mediates the long-term negative feedback gain of the postural system, which positively correlates to changes in the leaf fraction, BC_max_, and kappa but negatively correlates to the diameter and average eccentricity of all EEG sub-bands.

## Data Availability Statement

The raw data supporting the conclusions of this article will be made available by the authors, without undue reservation.

## Ethics Statement

The studies involving human participants were reviewed and approved by Institutional Review Board (IRB) at the National Cheng Kung University (NCKU) Hospital, Taiwan. The patients/participants provided their written informed consent to participate in this study.

## Author Contributions

I-SH and Y-CC designed and conceptualized the experiment. I-SH and C-CH performed analysis of EEG metrics. C-GZ collected data and preprocessed EEG data. Y-CC prepared the first draft. I-SH edited and finalized the manuscript. All authors contributed to the article and approved the submitted version.

## Conflict of Interest

The authors declare that the research was conducted in the absence of any commercial or financial relationships that could be construed as a potential conflict of interest.

## Abbreviations

Ave. Ecc: average eccentricity
BC_max_: the highest value of betweenness centrality
CD: critical point of displacement
CEA: confidence ellipse sway area
COP: center of pressure
D_s_: short-term diffusion coefficient
D_l_: long-term diffusion coefficient
EEG: electroencephalography
EOG: electrooculography
EC: eyes-closed
EO: eyes-open
FEF: frontal eye field
FIR: finite impulse response filter
H_s_: short-term scaling exponent
H_l_: long-term scaling exponent
ML: medial-lateral
MF: mean frequency
MST: minimum spanning tree
PLI: phase-lag index
RMS_AP_: root mean squared sway amplitude along the anterio-posterior direction
RMS_ML_: root mean squared sway amplitude along the medial-lateral direction
SampEn: sample entropy
SDA: stabilogram diffusion analysis.
